# A Scalable Method for Analysis and Display of DNA Sequences

**DOI:** 10.1371/journal.pone.0007051

**Published:** 2009-10-02

**Authors:** Lawrence Sirovich, Mark Y. Stoeckle, Yu Zhang

**Affiliations:** 1 Laboratory of Applied Mathematics, Mount Sinai School of Medicine, New York, New York, United States of America; 2 Program for the Human Environment, The Rockefeller University, New York, New York, United States of America; University of California, San Diego, South Africa

## Abstract

**Background:**

Comparative DNA sequence analysis provides insight into evolution and helps construct a natural classification reflecting the Tree of Life. The growing numbers of organisms represented in DNA databases challenge tree-building techniques and the vertical hierarchical classification may obscure relationships among some groups. Approaches that can incorporate sequence data from large numbers of taxa and enable visualization of affinities across groups are desirable.

**Methodology/Principal Findings:**

Toward this end, we developed a procedure for extracting diagnostic patterns in the form of indicator vectors from DNA sequences of taxonomic groups. In the present instance the indicator vectors were derived from mitochondrial cytochrome *c* oxidase I (COI) sequences of those groups and further analyzed on this basis. In the first example, indicator vectors for birds, fish, and butterflies were constructed from a training set of COI sequences, then correlations with test sequences not used to construct the indicator vector were determined. In all cases, correlation with the indicator vector correctly assigned test sequences to their proper group. In the second example, this approach was explored at the species level within the bird grouping; this also gave correct assignment, suggesting the possibility of automated procedures for classification at various taxonomic levels. A false-color matrix of vector correlations displayed affinities among species consistent with higher-order taxonomy.

**Conclusions/Significance:**

The indicator vectors preserved DNA character information and provided quantitative measures of correlations among taxonomic groups. This method is scalable to the largest datasets envisioned in this field, provides a visually-intuitive display that captures relational affinities derived from sequence data across a diversity of life forms, and is potentially a useful complement to current tree-building techniques for studying evolutionary processes based on DNA sequence data.

## Introduction

As Carl Woese first demonstrated over 30 years ago, the evolutionary history of organisms is embedded in their DNA [1]. The patterning of ancient divergences that led to present-day forms can be reconstructed by comparing homologous sequences from different organisms, thereby establishing a natural classification in the form of a Tree of Life that reflects evolutionary history [2]. Creating a Tree of Life for all organisms is a challenging task, given there are at least 1.7 million named species of extant plants and animals, plus innumerable fungi, protozoa, archaea and eubacteria [3].

The general approach to extracting phylogenetic information from DNA is the same as for morphologic analysis-arranging organisms in nested groups defined by synapomorphies, shared characters that represent a common evolutionary history [4] (Here and in the following the usage of group refers to taxonomic group.). Homologous gene sequences are aligned and the DNA characters at each site are used to infer evolutionary relationships, depicted as a branching tree diagram. In principle straightforward, in practice this is a computationally intensive procedure informed by complex models of nucleotide substitution [5]. The number of possible branching patterns increases logarithmically with the number of organisms [6], with the result that few trees with over 1,000 taxa have been generated (although see [7]). Alternatively, neighbor-joining (NJ), which uses distances rather than characters, can rapidly create phylogenies from large numbers of taxa with reasonable accuracy, although it is limited by saturation effects and restricted modeling of nucleotide substitution patterns [8]. The challenge of displaying evolutionary relationships among large numbers of organisms has stimulated new approaches to displaying and browsing trees [9], [10]. Phylogenetic trees assume branching evolutionary histories, limiting utility in some groups such as those with high rates of horizontal gene transfer. More generally, a tree diagram aims to express the temporal patterning of divergences and as such does not convey relative affinities among or within groups, such as might be due to positive or negative selection including convergent evolution. For these reasons, it is desirable explore complements to tree-based methods for analyzing and displaying DNA sequences from large numbers of organisms.

The methods presented in this paper apply to sequential biochemical data sets of general type. In the present exposition we specifically consider DNA sequences. We focus on the 648 nucleotide region of cytochrome *c* oxidase subunit I (COI) gene, employed as a standard “DNA barcode” for distinguishing animal species [11], and utilize records in Barcode of Life Database (BOLD) http://www.barcodinglife.org [12]. Broadly speaking, we aim to develop mathematically optimal procedures for extracting patterns and correlations from genetic databases. The main emphasis is on determining the correlation structure of existing life forms from biochemical data. From this we seek a rational depiction of the genetic “landscape” in terms of a reasonable metric. Possible past sequential states are not inferred. As shown later, the results of the present analysis have the potential for investigating evolutionary groups and affinities among the diversity of life forms.

## Results

**Figure 1:**
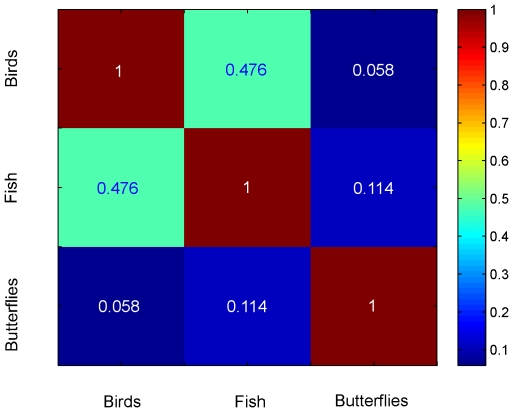
Correlation among group-level indicator vectors. A false-color map depicting correlations among indicator vectors 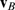, 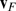, and 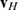 for COI sequences of birds, fish, and butterflies, respectively, is shown. The numerical correlation values are indicated.

The first example considers COI sequences with 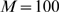 randomly drawn sequences from three BOLD projects representing different groups of animals: birds, fish, and butterflies. Indicator functions 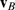, 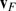, and 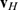 were constructed for these sequence sets as described in Material and Methods. Indicator vectors are a consequence of an optimization procedure which seeks a unit vector which is maximally correlated with a designated group, and simultaneously minimally correlated with the remaining groups under consideration. In general the results are collected together in the structure matrix
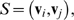
(1)the elements of which furnish the correlation coefficients between groups. A false color representation of the structure matrix provides a visual display of correlations among groups (Figure 1). These calculations indicated that fish and bird vectors were well correlated, as might be expected for two classes of vertebrates, and both were poorly correlated with the butterfly vector, consistent with more distant evolutionary relationships.

**Figure 2:**
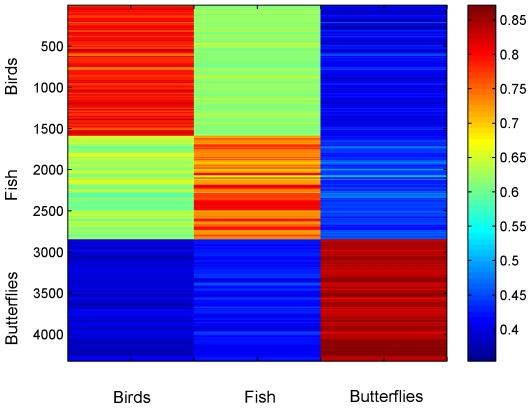
Correlation of test sequences with group-level indicator vectors. False-color map of 4,332 COI test sequences compared to the indicator vectors depicted in Figure 1. In all cases, the test sequences showed highest affinity with their respective group vector.

This indicator vector analysis was based on randomly choosing 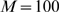 representatives for each of the base group matrices. This left a set of 4332 “test” sequences, i.e. those not used to construct indicator vectors (roughly 1600 bird, 1200 fish, and 1500 butterfly sequences). We then examined how well these test sequences were correlated with the indicator vectors. More specifically, each test sequence was translated into a vector as above, and correlations to the indicator vectors were determined. In all cases sequences from the test set were most highly correlated with the respective indicator vector for their group (Figure 2).

**Figure 3:**
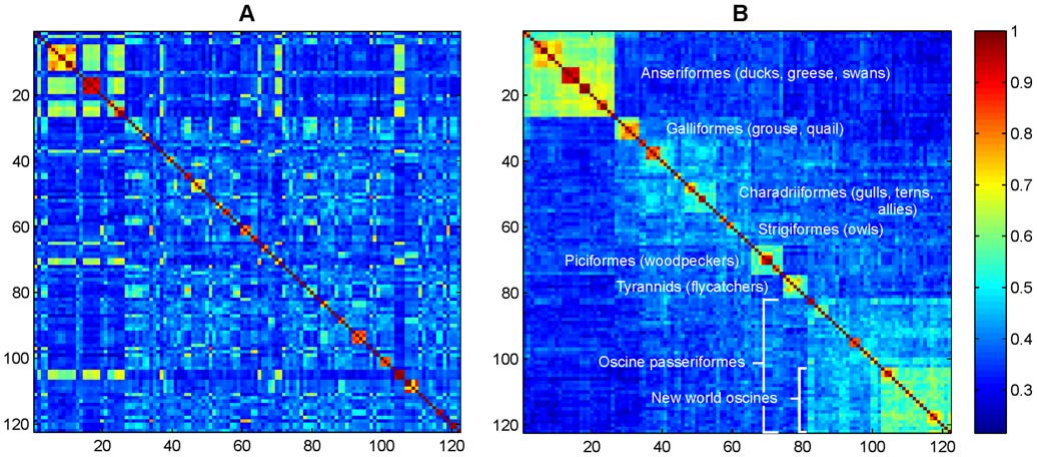
Correlation among species-level indicator vectors. A false color map depicting correlations among indicator vectors for 122 species of North American birds is shown in (A) where order is alphabetical by species name. Blocks of high correlation on the diagonal reflected affinity among species within genera. The large squares of highly-correlated birds in the upper left hand corner indicated close affinities among species in several genera of ducks and geese (*Aix, Anas, Athya, Branta, Bucephala*). In (B) the ordering follows established taxonomy, reflecting phylogenetic relationships [13].

A second example considers COI sequences of North American birds. Only those species for which at least 5 sequences were considered; 122 species were in this admissibility set. The resulting 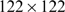 structure matrix, 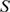, with vectors arranged alphabetically by species name is shown (Figure 3A). If instead the species are ordered according to accepted taxonomy [13] Figure 3B results, which shows harmony of the DNA-based indicator vector analysis with established phylogenetic relationships. The taxonomic ordering produces a relatively smooth mapping, with maximum correlation among neighboring species, and decorrelation among more distant species.

The test set for this framework contained 173 sequences and the 122 indicator vectors made correct species assignment in all cases.

## Discussion

In this paper we present a mathematical and graphical method for analyzing and displaying affinities among organisms based on DNA sequences. This approach has several desirable characteristics suggesting further study will be of interest. First, it is computationally efficient. Sequences are transformed into digital vectors and correlations among vectors are then calculated, with computations proportional to the number of input sequences (see Materials and Methods for details). Second, it is scalable in ability to incorporate large numbers of organisms, as above, and in that it can be applied to analyze correlations among sets of sequences at different taxonomic levels, as in the species and class examples shown. Third, it presents a visually-intuitive, condensed display of affinities among sequences in the form of a false-color map. A single figure can display information from at least 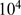 vectors, each of which can represent an unlimited number of sequences. Fourth, it provides a diagnostic approach in the form of “indicator vectors” which can be used to classify test sequences from unknowns. Finally, because the vectors preserve site-specific information, it is possible to recover the actual characters, both in individual and group-level vectors.

It is useful to consider our approach in the context of other methods for mining taxonomic information from DNA or protein sequences. In addition to those aimed at deeper phylogenetic reconstruction, these include tree-based techniques for species delimitation and statistical assignment of test sequences [14], [15]; non-tree search algorithms BLAST [16] and BLAT [17] which permit rapid, quantitative alignment of selected inputs to very large databases of tabulated sequences; and non-tree techniques for extracting diagnostic characters from sets of sequences [18], [19]. These are all character-based methods with a relatively narrow taxonomic focus. In contrast, our approach seeks macroscopic [20] relations among diverse groups of life forms. The digital transformation of sequential data employed is well suited to this sort of global analysis, whereas character-based search tools and diagnostics do not naturally lend themselves to this task, as it is problematical to generate a “mean” sequence representing a group of sequences using characters.

A potential application of this method might be in the construction of a hierarchical tree using the correlation matrix, although this possibility has not been examined. In the absence of a hierarchical tree, as in the analyses presented here, this method may be particularly useful for groups of organisms lacking established taxonomy including viral types and subtypes, and groups with reticulated evolutionary histories due to horizontal gene transfer, such as archaea and eubacteria. Importantly, the analysis as presented relies on existing taxonomic classification. It will be of interest to explore the potential for a bottom-up, sequence-based “classification” based on natural discontinuities in vector space, as suggested by Figure 3B.

The significance of relative affinities among indicator vectors is uncertain. In some cases, these were consistent with evolutionary relationships, as with finding of high correlation between birds and fish as compared to butterflies. In this comparison, there was greater correlation between butterflies and fish (albeit still very low) than between butterflies and birds (Figure 1). This latter observation might have a simple or trivial explanation, such as biases in AT vs. GC content or chance occurrence related to taxon sampling. On the other hand, it might be relevant that butterflies show greater affinity to fish, a relatively ancient lineage among vertebrates, than to birds, which arose more recently. Further study will help determine what sequence features underlie the patterns of correlation among indicator vectors and their possible biological significance. In this study we focused on COI because of the availability of a large number of sequences from diverse organisms. It will be of interest to compare results using other genes, individually or in combination, for which there is a large representation in public databases, e.g., nuclear genes for large and small subunit ribosomal RNA, ITS, chloroplast genes *rbcL* and *matK*, and mitochondrial genes other than COI. So far, there are sequences in GenBank from fewer than 160,000 of the 1.7 million named species of multicellular plants and animals, and genetic documentation of other eukaryotic lineages (fungi, protozoa) and the vast diversity of archaea and eubacteria is sparse. As representation grows, methods for exploring and displaying relationships among large numbers of sequences will be increasingly important. The mathematical and graphical approach presented here may be a useful addition.

## Materials and Methods

Nucleotide sequence data were downloaded from “Published Projects” section of BOLD as aligned fasta files. Although the amino acid sequence of COI is highly conserved across diverse forms of life, there are insertions of 1 or more amino acids in some species, necessitating the introduction of gaps into the alignment.

Examination of downloaded records revealed that terminal regions of the approximately 650 nucleotide segments had relatively high numbers of ambiguous and/or missing nucleotides, presumably reflecting incomplete sequencing runs. To reduce this uninformative “noise” we restricted attention to nucleotides in positions 100 through 600. This 501 nucleotide span contained 167 complete codons.

The aligned, trimmed sequences have been stored in MATLAB mat files which will be available along with relevant MATLAB code on our website.

### Data Transformation

Under the four letter genetic code a COI sequence in the above defined admissibility range translates to a vector of 501 components with entries A,T,C, and G. For quantitative purposes such a vector will be elaborated into vector of length 2004 having entries of 0 or 1 according to the convention
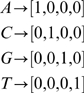
(2)


In schematic form
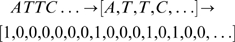
(3)


For the totality of sequences there were approximately 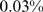 hyphen gaps, 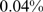 missing bases, 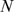, and 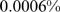 ambiguous missing data (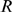, 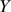, etc.). Gaps were initially encoded as 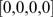. Missing bps were encoded either as modal or as average values. Neither produced any significant effect. In addition there were about 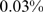 bps which were misaligned, and many of these were corrected manually. This also proved to be virtually insignificant. Thus each sequence has a unique representation in the chosen vector space, and a Hamming distance [21] may be applied immediately.

### Indicator Vectors

We considered the existence of a distinguishing vector which is indicative of a specific group of organisms, determined on the basis of its contrast with vectors of other sets of organisms. A narrow, but perhaps illuminating view of the procedure is that we seek an objective and automated algorithm for inclusion/exclusion of a sequence as a member of a specific group within a set of groups, by means of correlations with the sought after indicator vectors.

The process of constructing indicator vectors can be carried out following the levels of the traditional hierarchical taxonomic classification, e.g. phyla, classes, species, etc. However the procedure as presented is robust and can be applied across non-traditional boundaries. In the first example, we considered COI sequences for three groups of animals which we informally titled “birds”, “fish,” and “butterflies.” COI sequences utilized for these three groups were drawn from “Published Projects” section in BOLD [12], namely “Birds of North America -Phase II [22] “Barcoding of Canadian freshwater fishes” [23], and “Hesperidae of the ACG 1” [24]. The second example considers the species contained within the North American bird project. In all cases we only consider sequences with sufficient length, and we exclude those containing excessive blanks.

The range 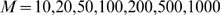 for the Birds/Fish/Butterflies case, was examined for efficiency and timing on the basis of Matlab code and a modest desktop machine. No test set errors occurred for 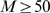. Computational times varied roughly linearly from 7 sec at 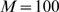 to 24 sec at 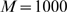. The discrepancy in calculating indicator vectors on passing from 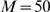 to 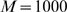 is less than 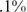. In addition we performed a trial calculation involving 12 groupings with 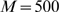, which took roughly 30 sec.

### Mathematical Methods

Consider a collection of 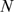 groups 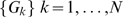. Explicitly in the first example we consider 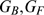 and 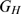 the groups of (North American) Birds, (Canadian) Fish and (butterflies) Hesperidae. For each group 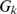 a fixed number 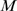 of representative sequence vectors 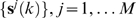 are selected at random and the base group matrices
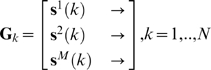
(4)are formed. Thus for the first example case we form 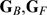 and 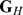.

For the 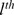 group we seek its indicator vector 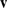, defined to be of unit length
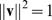
(5)and such that it extremizes the criterion functional
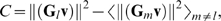
(6)where 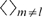 is the average over all 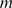 except 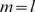, is maximal. In more detailed form
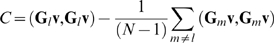
(7)where 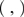 indicates the appropriate inner product.

In words the optimization seeks the indicator vector 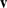 which, if all within-group members were identical, would have a unit correlation coefficient with the 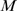 member sequences of 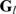 and a zero correlation coefficient with members of all other groups 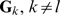. (A similar approach has been used to reveal cortical organization contained in optical imaging: [25], [26].)

A standard variational argument leads to the eigenvalue problem
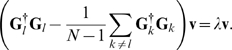
(8)


It is at least intuitively obvious that the maximal eigenvalue is positive, and under reasonable hypotheses this may be proven. Thus the eigenvector corresponding to the maximal eigenvalue yields the indicator vector for the 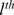 group. This and the eigenvalue are denoted by
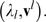
(9)


This procedure is carried out successively for each group.

The residual sequences not used in constructing the base matrices 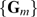 then furnish a test set for evaluating the accuracy of the procedure.
